# Using selection tests to hire autistic employees: An exploration of methods

**DOI:** 10.1371/journal.pone.0356539

**Published:** 2026-08-18

**Authors:** Christopher E. Whelpley, Tyler Fezzey, John H. Batchelor, Jeff A. Weekley

**Affiliations:** 1 Management and Entrepreneurship, School of Business, Virginia Commonwealth University, Richmond, Virginia, United States of America; 2 Department of Business Administration, Lewis Bear Jr. College of Business, Pensacola, Florida, United States of America; 3 Retired; Taipei Veterans General Hospital, TAIWAN

## Abstract

A persistent barrier to employment for autistic individuals is the personnel selection process. Although prior research demonstrates that traditional employment interviews disadvantage autistic applicants relative to neurotypical applicants, far less is known about whether commonly used alternative selection methods reduce or reproduce such disparities. Drawing on a sample of autistic and neurotypical adults, this study examines subgroup differences across three widely used selection tools: general mental ability (GMA) tests, personality inventories, and situational judgment tests (SJTs). We assess subgroup differences using standardized mean comparisons and further examine predictors of SJT performance using multivariate regression analyses. The results indicate that all three selection methods produce meaningful subgroup differences that are unfavorable to autistic respondents. Moreover, autistic respondents scored significantly lower on the SJT even after controlling for GMA, personality traits, gender, and age. Together, these preliminary findings suggest that commonly used selection tools, often promoted as objective alternatives to interviews, may nonetheless result in adverse impact for autistic applicants if used in hiring decisions. We discuss implications for personnel selection research, the design of inclusive hiring systems, and the need to develop assessment methods that better capture job-relevant strengths among neurodiverse applicants.

## Introduction

Improving selection outcomes for individuals on the autism spectrum is a pressing social and academic issue [1]. Estimates suggest that up to 80% of autistic individuals are unemployed or underemployed, underscoring the severity of employment barriers facing this population [2]. As diagnoses of autism spectrum disorder (ASD) become more prevalent, alongside earlier identification and interventions, finding fair and effective methods for hiring autistic individuals is increasingly important for organizations, policymakers, and scholars [3]. Correspondingly, a variety of academic domains have called for research examining the employment hurdles faced by individuals on the spectrum (e.g., [4–6]), as well as for management research that generates meaningful impact beyond academia [7,8].

From a human capital and strategic management perspective, autistic employees may represent an underutilized source of competitive advantage. Building on the social model of disability, scholars have argued that employers should view autism through the lens of diversity rather than as a deficit and recognize the distinctive strengths that autistic individuals can bring to organizations [9,10]. Though heterogeneous as a population, autistic individuals are often characterized as possessing strong pattern recognition abilities, the capacity for sustained concentration, and deep expertise in areas of interest, attributes that may be particularly valuable in knowledge intensive and technical roles [11]. Empirical and practitioner-oriented research further suggests that these strengths are recognized and valued by managers in the workplace [12]. When SAP launched its neurodiverse specific hiring program, for example, the organization explicitly framed autistic talent as a strategic resource, emphasizing that “innovation comes from the edges” [13]. In line with broader strategic human capital theory, such distinctive skills and perspectives can function as organizational resources that contribute to sustained competitive advantage [14,15].

Despite these potential advantages, the personnel selection process itself remains a central barrier to employment for autistic individuals. How autistic applicants perform across different selection methods remains poorly understood as the extant literature primarily focuses on interview performance alone [16]. Prior research suggests that traditional interviews result in adverse outcomes for autistic applicants relative to neurotypical applicants, even when job-relevant qualifications are comparable. These effects appear to be driven primarily by differences in social communication, nonverbal behavior, and interpersonal impression management rather than substantive differences in ability [17]. Qualitative evidence further indicates that autistic applicants may find interviews particularly challenging and prefer written or structured assessment formats that reduce social ambiguity [18].

In response to these concerns, both scholars and practitioners have suggested that alternative selection methods may reduce “noise” not related to job performance and promote more equitable hiring outcomes for autistic applicants. Anecdotal and practitioner evidence points to initiatives at companies such as Microsoft and SAP, which have implemented extended or modified interview processes for applicants on the autism spectrum [1,19]. However, such programs account for a relatively small proportion of the total workforce and have yet to have create an impact at societal level leading [20]. More critically, there is little empirical evidence evaluating whether commonly used employment tests, often proposed as objective alternatives to interviews, would reduce adverse impact for autistic applicants in a selection setting. Given that autism is characterized by persistent differences in social communication and interaction, as well as restricted or repetitive patterns of behavior and interests [21,22], it remains unclear whether selection tools that rely on normative comparison, social judgment, or self-referential assessment inadvertently disadvantage autistic individuals. This uncertainty is compounded by the heterogeneity of ASD, which spans a wide range of functional levels and lived experiences, from individuals whose diagnosis is not readily apparent to others for whom daily functioning is substantially impacted [4,23].

The present study starts to address this shortcoming by examining how autistic and neurotypical individuals perform across three widely used personnel selection methods: general mental ability (GMA) tests, personality inventories, and situational judgment tests (SJTs). Specifically, we compare subgroup differences across these assessment tools to evaluate whether they may reduce or reproduce the adverse impact observed in traditional interviews. We also explore whether differences in SJT performance persist after accounting for GMA and personality traits. In doing so, this study makes several contributions. First, using a sample that approximates an applicant pool by recruiting autistic and neurotypical respondents that either had an employment history from which to draw or were currently employed, we extend research on autism and employment beyond interviews to other components of selection systems. Second, we inform selection and inclusion research by evaluating whether commonly used “objective” assessment tools function in a valid manner in our neurodiverse sample. Third, we provide organizations with evidence-based guidance regarding the unintended consequences of widely accepted hiring practices. Collectively, these contributions advance the understanding of how personnel selection systems shape access to employment for autistic individuals and inform efforts to design more inclusive hiring practices.

## Theoretical background

Companies can create competitive advantage if they attract and hire productive individuals overlooked by other employers [24]. Hiring practices have been a central concern in both practice and research for many years [25]. How selection methods might harm different neurodiverse subpopulations has been a similar concern among a variety of stakeholders, including companies, autistic applicants, their families, and broader society. This harm can be measured through subgroup differences in selection methods that, if used, could result in adverse impact [26,27]. Generally speaking, adverse impact is examined through the lens of demographic fault lines, most often operationalized as race and gender [28]; however, as notions of diversity expand, evolve, and diminish so too does our need to understand how selection methods may advantage or disadvantage groups of applicants not previously considered, such as the neurodiverse population [29]. Across selection methods, adverse impact may arise not only from differences in job-relevant ability but also from mismatches between the cognitive, interpersonal, and evaluative demands in assessment tools and applicants’ perceptions, interpretations, and responses to those demands. For autistic applicants, characteristics associated with social communication, interpersonal inference, and self-referential comparison may interact with these assessment demands in ways that disadvantage performance, even when underlying job-relevant skills are present.

Research has found both qualitative and quantitative support that interviews can disadvantage autistic applicants [16,18]. For example, Maras and colleagues [16] found that the content of questions can influence interview outcomes. Specifically, when interviewers broke down broad questions into smaller ones and probed for specific examples, autistic individuals reduced the performance gap relative to neurotypical interviewees. Building on this finding, Whelpley and May [17] found that autistic applicants scored lower on interview performance than neurotypical interviewees in a simulated video setting. However, when the same interviews were converted to written transcripts and read rather than viewed, autistic interviewees outperformed neurotypical individuals, leading to the conclusion that social and visual cues, rather than substantive differences, contributed to lower evaluations for autistic individuals. Thus, the adverse outcomes from interviews may be addressed by implementing alternative selection methods that provide meaningful validity for employers while also offering more objective evaluations of applicant potential, independent of the social cues presented in interviews. Among these employment tests, personality inventories, GMA tests, and SJTs have historically been used as hiring methods with appropriate levels of criterion validity [25,30], and varying levels of adverse impact [27]. Next, we briefly explore these selection methods and present research questions concerning the use of each method to select autistic applicants relative to neurotypical applicants.

### Personality inventories

The Five-Factor model of personality has been used to select applicants for over three decades [31]. It is generally believed that using personality facets to select employees is valid for predicting job performance [32] and has lower documented adverse impact than other methods [33]. However, it is unclear how autistic applicants would score on these selection tests. To date, few personality studies have addressed groups of autistic job applicants specifically [34,35]. The limited personality research that exists addressing autistic individuals has suggested that there are differences between those with and without higher levels of expression of ASD characteristics [36]. Specifically, those with more expressive autistic traits are less extroverted, less open to new experiences, less conscientious, less agreeable, and more neurotic. However, this research did not explore personality in the context of selection.

Notably, many personality inventory items require respondents to interpret social norms, evaluate themselves relative to others, or infer how their behavior is perceived in interpersonal contexts. Given documented differences in social cognition and self-referential processing associated with autism, such features of personality assessment may differentially shape how autistic applicants respond to these instruments. It may also be the case that autistic individuals who are actively employed or have an employment history differ meaningfully from other subgroups with an ASD diagnosis. Thus, we seek to address the following research question:

*RQ1: What subgroup differences exist between autistic respondents and neurotypical respondents in terms of the Five-Factor model of personality?*

### General mental ability tests

General mental ability is one of the most valid predictors of job performance across domains and has decades of research supporting its use as a selection construct [25]. However, GMA tests can result in adverse impact when used in the absence of other measures for some subgroups of applicants [27,37]. To date, nationally representative samples of autistic individuals exploring the distribution of GMA across the spectrum have not been performed. However, initial research suggests that the distribution is likely flatter than that of the neurotypical population, with more individuals on the tails [38]. The same research found that autistic individuals are overrepresented at high GMA levels in comparison with non-autistic samples. Despite these insights, research has not examined how GMA tests function specifically for autistic job seekers applying through traditional hiring systems. Consequently, it remains unclear how the use of GMA tests as a selection method influences the hiring of autistic applicants. Thus, we seek to answer the following:

*RQ2: What subgroup differences exist between autistic respondents and neurotypical respondents in terms of general mental ability tests?*

### Situational judgment tests

Situational judgment tests are a valid selection method that measure a variety of underlying characteristics [39]. In SJTs, applicants read a scenario in which they are presented with a social situation and asked to evaluate it. One theory that explains the validity of SJTs is the tenet of behavioral consistency, whereby test responses translate into decisions made in the work environment [40]. SJTs are considered a selection method that measures an assortment of constructs, including both personality and GMA [41]. Based on incremental validity, it is proposed that SJTs also measure judgment and/or social intelligence [39,42]. To the extent that SJTs capture social judgment or interpersonal inference in addition to job-relevant decision-making, they may systematically disadvantage autistic applicants independent of cognitive ability or personality traits. This inference aligns with diagnostic criteria for autism, which include differences in social-emotional understanding and relationship interpretation [21]. However, research has not explored SJTs as a tool for hiring autistic applicants. Accordingly, we pose the following research question:

*RQ3: What subgroup differences exist between autistic respondents and neurotypical respondents in terms of SJT scores?*

## Methods

### Participants

The research protocol, including the consent, was reviewed and approved by the internal review board at Virginia Commonwealth University; consent was collected electronically (IRB Study: HM20021533). We recruited two independent samples through Amazon’s MTurk. MTurk is widely regarded as a means of crowd-sourcing unique respondents and has been used by other scholars to recruit neurodiverse participants [43,44]. Additionally, studies have suggested that the use of MTurk provides an appropriate distribution on measures such as general mental ability [45]. The first sample consisted of non-autistic individuals and included 99 respondents with an average age of 37.8 years (47.5% female). All but 5 respondents were currently employed and the others had an employment history to draw on. The second sample included 107 autistic respondents, with an average age of 35.1 years (41.1% female), and the entire sample was currently employed. In line with the diagnostic criteria from the DSM-5, respondents had to indicate they had received a diagnosis of autism spectrum disorder, Asperger’s syndrome, or pervasive developmental disorder not otherwise specified. Given the high unemployment among autistic individuals, we felt that using employed or previously employed respondents would yield a sample more like those applying to jobs in actual organizations. Both sets of respondents were over the age of 18 and their own legal guardian. By requiring our respondents to be legally responsible for themselves, our sample better reflects individuals who would be seeking competitive employment in a traditional selection context. We also incorporated best practices from Aguinis and colleagues [46], including two foil questions and a qualitative question, to improve the quality of our responses and detect non-attentive respondents. Based on the responses, we removed seven individuals from each sample, for a total of 14 removed, bringing the sample sizes to the aforementioned 99 and 107 for neurotypical and autistic respondents, respectively. Prior to receiving the consent form, all participants had to indicate that they were legally responsible for themselves. Again, individuals from the autistic sample had to indicate that they had received a diagnosis in line with the definition provided by the DSM-5 manual [21]. Because all respondents were legally responsible for themselves and had employment histories from which to draw, no further information regarding service needs was collected. Responses were collected from June 6, 2022, to June 9, 2022. The dataset can be found here: Autism & SJTs - Public File.

### Measures

***Personality Inventory.*** We measured the Five-Factor model using the 50-item International Personality Item Pool scale [47,48]. Each facet (agreeableness, conscientiousness, emotional stability, extraversion, and openness) was measured using 10 items each. In our neurotypical sample, the reliabilities for agreeableness, conscientiousness, emotional stability, extraversion, and openness were 0.82, 0.75, 0.86, 0.79, and 0.71, respectively. In our autistic sample, the reliabilities for agreeableness, conscientiousness, emotional stability, extraversion, and openness were 0.10, 0.38, 0.76, 0.43, and 0.20, respectively. We further discuss these lower-than-expected reliabilities in the limitations section.

***General Mental Ability.*** We used the 16-item International Cognitive Ability Resource (ICAR-16) to test general mental ability [49]. The ICAR-16 is a freely available nonproprietary measure that has been validated with field research [50]. The reliability for the GMA test was 0.58 and 0.51 for the neurotypical and autistic samples, respectively. Please note that the ICAR-16 includes items from different facets of intelligence and thus is a multidimensional measure. It has also been noted that the ICAR-16 has lower reliability than measures with more items, but it is still a robust measure of general mental ability [51,52]. As such, it is unclear how coefficient alpha should be interpreted in our sample, or what we would expect to find [53].

***Situational Judgment Test.*** Our situational judgment test was designed to select workers in a customer service job. This proprietary test was validated in the field and provided to the authors but has yet to be used in other publications. This SJT is currently owned by a corporation and is not available to the public. It includes 12 stems, each with five response options. The respondents were asked to choose both the best and worst response options, resulting in a total of 24 responses. Scores were awarded 1 point for a correct response and deducted 1 point for an incorrect response (i.e., they chose the worst response when asked to choose the best).

## Results

We present the correlation matrices in Tables 1 and 2 for the autistic and neurotypical candidates, respectively. We include the mean differences and Cohen’s *d* for the Five-Factor model in Table 3 and in Table 4 for GMA and SJT. In response to RQ1, which explores differences in personality between autistic and neurotypical respondents, we find meaningful differences between our groups. Specifically, findings indicate that neurotypical respondents demonstrated significantly higher agreeableness (*d* = −0.59), conscientiousness (*d* = −0.69), and emotional stability (*d* = −0.98) (see Table 3). There were non-significant differences between extraversion (*d* = −0.17) and openness (*d* = −0.23), both of which were again higher in our neurotypical sample.

**Table 1:** Correlation matrix – Autistic respondents.

	Mean	Standard Deviation	Gender	Age	SJT	GMA	Agreeableness	Conscientiousness	Emotional Stability	Extraversion
Gender	0.59	0.49								
Age	35.14	7.50	0.13							
SJT	0.57	3.49	−0.07	0.10						
GMA	4.73	2.35	−0.37**	0.03	0.21*					
Agreeableness	3.36	0.34	0.08	0.07	0.04	0.06				
Conscientiousness	3.36	0.36	0.01	0.08	0.34**	−0.07	0.43**			
Emotional Stability	2.54	0.64	−0.28**	0.13	0.38**	0.23*	0.31**	0.51**		
Extraversion	2.97	0.45	0.04	0.15	−0.19*	−0.02	0.31**	0.19	0.19	
Openness	3.59	0.37	0.04	−0.02	0.09	0.08	0.59**	0.52**	0.16	−0.04

Note: Male = 1, Female = 0; * *p* < .05, ** *p* < .01.

**Table 2:** Correlation matrix – Neurotypical respondents.

	Mean	Standard Deviation	Gender	Age	SJT	GMA	Agreeableness	Conscientiousness	Emotional Stability	Extraversion
Gender	0.53	0.50								
Age	37.84	10.60	−0.01							
SJT	4.24	5.14	0.10	0.25*						
GMA	5.96	2.42	−0.11	0.08	0.33**					
Agreeableness	3.67	0.68	−0.16	0.13	0.15	0.05				
Conscientiousness	3.69	0.61	−0.03	0.16	0.32**	0.13	0.50**			
Emotional Stability	3.29	0.87	−0.07	0.10	0.39**	0.28**	0.54**	0.56**		
Extraversion	3.07	0.70	−0.09	0.02	−0.28	0.04	0.41**	0.25*	0.26*	
Openness	3.70	0.57	−0.03	0.03	0.11	0.17	0.61**	0.47**	0.35**	0.47**

Note: Male = 1, Female = 0; * *p* < .05, ** *p* < .01.

**Table 3:** Personality means and Cohen’s *d.*

Personality Means and Cohen’s *d*										
	Agreeableness		Conscientiousness		Emotional Stability		Extraversion		Openness	
	Autistic	Neurotypical	Autistic	Neurotypical	Autistic	Neurotypical	Autistic	Neurotypical	Autistic	Neurotypical
Mean	3.36	3.67	3.36	3.69	2.54	3.29	2.97	3.07	3.59	3.70
S.D.	0.34	0.68	0.36	0.61	0.64	0.87	0.45	0.70	0.37	0.57
Cohen’s *d*	−0.59**		−0.69**		−0.98**		−0.17		−0.23*	
95% C.I.	(−0.87, −0.31)		(−0.97, −0.41)		(−1.27, −0.69)		(−0.45, 0.10)		(−0.51, 0.04)	

Note: * *p* < .05, ** *p* < .01.

**Table 4:** SJT & GMA Means and Cohen’s *d.*

SJT & GMA Means and Cohen’s *d*				
	SJT		GMA	
	Autistic	Neurotypical	Autistic	Neurotypical
Mean	0.57	4.24	4.73	5.96
S.D.	3.49	5.13	2.35	2.42
Cohen’s *d*	−0.84**		−0.52**	
95% C.I.	(−1.13, −0.56)		(−0.79, −0.24)	

Note: * *p* < .05, ** *p* < .01.

In response to RQ2 and RQ3, we found significant differences in both GMA and SJT scores (see Table 4). The mean score for the GMA test was 5.96 in the neurotypical sample and 4.73 in the autistic sample. Cohen’s *d* between these two samples was 0.52, consistent with a large effect size. It is important to note that research has not found differences between autistic and neurotypical individuals in GMA at a population level and thus our finding is likely specific to our sample [38]. The neurotypical respondents averaged a score of 4.24 on the SJT, whereas the autistic respondents averaged a score of 0.57 (Please note that because you can lose points for choosing the worst response as the best response, and vice versa, the distribution included negative scores in both of our samples.). Cohen’s *d* was 0.84 for the SJT, consistent with a large effect size.

We next explore some of the correlations between the variables that we found interesting but did not include in our research questions. An important correlation to note is between GMA and SJT. As mentioned, previous research has found that SJTs are cognitively demanding as they measure aspects of GMA [30]. Indeed, when we examine the correlation between GMA and the SJT in our neurotypical sample, we find a correlation of 0.33. In our neurodiverse sample, the correlation between GMA and SJT is 0.21. Given this finding, we asked what variables predict SJT score and used multivariate regression to explore this. Specifically, we built a multivariate regression model (see Table 5) controlling for gender, age, GMA, and personality. Our results indicate that after including these controls, being autistic still had a significant negative impact on the SJT score. We also note that other control variables could have been included in model (e.g., job tenure) and that these results should be treated as preliminary, which is further discussed in the limitations.

**Table 5:** Standardized regression coefficient and variance explained in SJT score.

	Model 1	Model 2	Model 3	Model 4
Gender	0.00	0.08	0.11*	0.11
Age	0.24**	0.20**	0.14*	0.13*
GMA		0.34**	0.22**	0.20**
Agreeableness			0.02	0.00
Conscientiousness			0.24**	0.22**
Emotional Stability			0.36**	0.31**
Extraversion			−0.36**	−0.35**
Openness			−0.02	0.00
Autism Diagnosis				−0.15*
R	0.24	0.41	0.67	0.68
R^2^	0.06	0.17	0.45	0.47
Abs. Change (R^2^)		0.11	0.29	0.02
Sig. of Change		<.01	<.01	0.01

Note: * *p* < .05, ** *p* < .01.

Note: Male = 1, Female = 0.

## Discussion

The present study examined whether commonly used personnel selection tools (personality inventories, GMA tests, and SJTs) could produce differential outcomes for autistic and neurotypical respondents in a sample that approximated job applicants. Across all three selection methods, autistic respondents scored lower on average than neurotypical respondents, indicating that subgroup differences unfavorable to autistic applicants are not confined to interview-based selection methods. These results, though preliminary, challenge the assumption that shifting away from interviews toward ostensibly more objective assessments necessarily improves equity in hiring outcomes for neurodiverse applicants. However, it is important to note that due to low reliabilities in personality for our autistic sample, these results are nascent in the field of study and descriptive in nature. The finding that differences in SJT performance persisted even after controlling for gender, GMA, age, and personality traits is important because previous research has not found differences in GMA between autistic and neurotypical individuals at a population level indicating this difference is likely idiosyncratic to our sample [38]. However, after controlling for differences in cognitive ability and personality dimensions we still found autism to be a negative predictor of SJT score. This is consistent with the earlier theoretical framing emphasizing that subgroup differences may arise from mismatches between the demands of the assessment tools and applicants’ perceptions, interpretations, and responses to evaluative situations. Selection methods like SJTs that implicitly rely on social judgment, interpersonal inference, or normative interpretation may disadvantage autistic applicants despite possessing job-relevant skills. Together, these findings extend prior research on autism and employment by demonstrating that adverse impact for autistic applicants may occur if widely used components of modern selection systems are used. Whereas previous work has documented bias in interviews, the present study suggests that alternative selection tools may reproduce similar disparities through different mechanisms.

### Theoretical implications

This research contributes to personnel selection theory by extending the concept of adverse outcomes beyond traditional demographic categories and interview-based assessments to neurodiverse populations and commonly used employment tests. Although subgroup differences have historically been examined primarily with respect to race and gender [28], our findings underscore the importance of considering how selection tools function for groups defined by cognitive and social characteristics. In doing so, this study aligns with emerging calls to broaden research in management to include neurodiversity as a theoretically meaningful dimension [29]. Our results suggest that common selection methods may result in adverse outcomes and these outcomes can occur when assessment methods embed cognitive, interpersonal, or evaluative demands that differentially interact with applicants’ information and social processing. In turn, the results point to the need for research to explore how selection tools viewed as neutral indicators of performance could nonetheless disadvantage autistic applicants.

The findings related to SJTs are particularly informative. Although SJTs are often conceptualized as low-fidelity simulations that capture job-relevant decision-making through behavioral consistency [40], they may also draw on constructs such as judgment and social intelligence [39,42]. To the extent that SJTs require applicants to infer social norms or interpret interpersonal dynamics, they may systematically disadvantage individuals whose social-cognitive processing differs from the neurotypical norm. Importantly, this interpretation does not imply a lack of job-relevant capability among autistic applicants but rather may point to construct contamination in selection methods if social inference is not central to job performance. Finally, this work contributes to the autism and employment literature by demonstrating that subgroup differences may persist even among employed autistic adults, suggesting that selection barriers could exist even for those with employment experience. This finding underscores the importance of future research examining selection systems as ongoing filters that shape neurodiverse individuals’ access to work opportunities.

### Practical implications

From a practical perspective, the present findings carry implications for organizations seeking to design inclusive hiring systems. Although interviews are often criticized for disadvantaging autistic applicants, our results suggest that simply replacing interviews with common employment tests may not resolve inequities in selection outcomes. Personality inventories, GMA tests, and SJTs (all widely used and often viewed as objective) were each associated with subgroup differences unfavorable to autistic applicants in this study. Accordingly, organizations should exercise caution in assuming that standard selection tools automatically promote fairness for neurodiverse applicants. In particular, selection methods that rely on social judgment, self-referential comparison, or implicit interpersonal norms may inadvertently screen out qualified autistic candidates when those skills are not central to job performance. Rather than abandoning structured assessment altogether, organizations may need to reconsider what is being assessed and how closely those assessments align with essential job requirements.

These findings also suggest that greater emphasis on job-relevant, task-based assessments, such as work samples or skill demonstrations, may hold promise for reducing adverse impact while preserving predictive validity. Indeed, practitioner-oriented autism hiring programs that focus on project-based evaluation or direct skill demonstration may offer valuable insights for rethinking selection system design. Specifically, the results highlight the importance of treating validity as a design problem rather than a tool-selection problem, requiring careful consideration of how assessment demands interact with applicant characteristics.

### Limitations and future research

The present study is subject to several limitations that also point to important directions for future inquiry. First, the reliability of the personality measures in the autistic sample fell below commonly accepted thresholds [54]. This warrants caution in interpreting subgroup differences on personality inventories and underscores broader concerns about the suitability of standard personality assessments for neurodiverse populations. Many personality inventory items implicitly rely on social comparison or on interpreting interpersonal norms (e.g., “Am the life of the party,” “Sympathize with others’ feelings”), which may be construed differently by autistic and neurotypical respondents. These measurement challenges suggest that commonly used personality inventories may not function equivalently across neurodiverse groups, limiting their utility as fair selection tools. Further, additional control variables could have been added to the hierarchical model, which may have shifted our results (e.g., organizational tenure or educational attainment). Future research should examine measurement equivalence and develop or adapt personality assessments that better capture job-relevant tendencies without relying on social or normative framing that may disadvantage autistic applicants.

Second, the SJT used in this study was developed for a customer service context and validated using a predominantly neurotypical workforce. It is therefore possible that the observed subgroup differences reflect a misalignment between the interpersonal demands in this particular SJT and the types of roles for which many autistic individuals may be best suited. SJTs developed for other occupational contexts, (e.g., those emphasizing technical, analytical, or procedural work), may yield different patterns of results. Future research should systematically examine how SJT content, response format, and underlying construct emphasis (e.g., social judgment versus procedural knowledge) influence subgroup differences. More broadly, researchers should assess whether SJTs, GMA tests, and personality inventories demonstrate criterion-related validity for autistic employees, that is, whether performance on these assessments predicts meaningful outcomes such as job performance, retention, or well-being for autistic workers.

Third, our findings raise important questions about the design of selection systems generally. Popular press accounts frequently highlight autism-focused hiring initiatives at organizations such as Microsoft and SAP [1,19], which often rely on project-based evaluation and direct skill demonstration rather than traditional interviews or standardized tests. While the scalability of these programs has been questioned, they nonetheless point to alternative approaches that merit rigorous academic study. In particular, practices from Microsoft and other companies may overlap conceptually with assessment centers, which emphasize job-relevant simulations and work samples [55]. Recent research suggests that assessment centers can be effective when they prioritize task-based performance over interpersonal impression management [56], potentially making them better suited for evaluating autistic applicants who possess strong job skills but differ in social communication styles. Future research should explore whether assessment centers or other work sample related methods reduce adverse impact while maintaining validity for neurodiverse populations.

Finally, the present study is constrained by its sample. Although MTurk is an established method for recruiting autistic participants in organizational research [12,44], the generalizability of our findings depends on the extent to which our respondents resemble actual applicant pools. We mitigated this concern by restricting participation to individuals who self-identified as autistic and had employment histories, thereby approximating an active labor market population. Nonetheless, future research should replicate these findings using field samples of job applicants and employees, as well as across industries and occupational levels. Further, it is possible that people misclassified themselves either accidentally or to participate in the research. Future research could use longitudinal designs that track selection outcomes through hiring, onboarding, and performance would be especially valuable for understanding how early selection decisions shape longer-term employment trajectories for autistic individuals.

## Conclusion

There is a growing need to identify and implement valid hiring methods for autistic applicants that can scale across organizations as diagnoses of autism spectrum disorder continue to increase. The present study provides initial evidence that several widely used personnel selection tools are associated with subgroup differences unfavorable to autistic applicants suggesting that, if used in selection, adverse impact may not be confined to interviews alone. To our knowledge, this research represents the first systematic comparison of autistic and neurotypical applicants’ performance across multiple core components of selection systems, thereby contributing to both personnel selection theory and the emerging literature on neurodiversity at work. Importantly, these findings do not imply a lack of job-relevant capability among autistic applicants; rather, they highlight how commonly used assessment tools may disadvantage qualified individuals when their embedded demands are misaligned with how applicants perceive and respond to evaluative situations. We hope that future research builds on this work to develop and validate selection approaches that better align assessment methods with job specific requirements while minimizing subgroup differences, ultimately supporting more equitable and effective hiring of neurodiverse talent. We also suggest that readers interested in this area explore work by Whelpley & May, 2022, Ezerins et al., 2024, and Maras et al., 2021.
